# New Insights Into Immune Cell-Derived Extracellular Vesicles in Multiple Sclerosis

**DOI:** 10.3389/fneur.2018.00604

**Published:** 2018-08-13

**Authors:** Maria Blonda, Antonella Amoruso, Tommaso Martino, Carlo Avolio

**Affiliations:** Department of Medical and Surgical Sciences, University of Foggia, Foggia, Italy

**Keywords:** extracellular vesicles, microvesicles, exosomes, Multiple Sclerosis, biomarkers of neurodegenerative disorders

## Abstract

Extracellular vesicles (EVs) are small vesicles including microvesicles and exosomes which differ in their distinct size, density, biogenesis, and content. Until recently, EVs were considered as simply scrap products. Nowadays, they are engendering huge interest and their shedding plays a well-recognized role in intercellular communication, not only participating in many physiological processes, but also suspected of being involved in the pathogenesis of many diseases. The present review aims to summarize the latest updates on immune cell-derived EVs, focusing on the current status of knowledge in Multiple Sclerosis. Significant progress has been made on their physical and biological characterization even though many aspects remain unclear and need to be addressed. However, it is worth further investigating in order to deepen the knowledge of this unexplored and fascinating field that could lead to intriguing findings in the evaluation of EVs as biomarkers in monitoring the course of diseases and the response to treatments.

## Definition and characteristics of EVs

Cells use different mechanisms to communicate with each other, such as direct contact or secretion of signal molecules. Recent studies suggest the existence of a previously unknown communication route based on the release into the extracellular space of a special cargo called extracellular vesicles (EVs). They include both microvesicles (MVs), also known as ectosomes, and exosomes (EXOs), which differ in their size, density, biogenesis and content. MVs are defined as small vesicles (100–1,000 nm) budding specifically from the surface of the plasma membrane and are expelled into the extracellular milieu. EXOs (40–100 nm) are lower membrane vesicles coming from inward budding of multivesicular bodies that, by fusing with the plasma membrane, release their contents into the extracellular compartment (1). The characteristic features of the two extracellular vesicle populations are summarized in Table 1.

**Table 1:** Main characteristics of different type of EVs.

**Characteristics**	**EVs**	
	**MVs**	**EXOs**
Size	100–1,000 nm	40–100 nm
Shape	Irregular	Round
Density	• Sucrose: 1.16 g/ml • Iodixanol: 1.18–1.20 g/ml	• Sucrose: 1.13–1.21 g/ml • Iodixanol: 1.10–1.12 g/ml
Biogenesis	Budding directly from the cell plasma membrane	Released from multivesicular bodies fused with the plasma membrane at the end of endocytic pathway
Lipid composition	Phosphatidylserine, cholesterol	Cholesterol, ceramide
Content	mRNA, miRNA, non-coding RNAs, dsDNAs, cytoplasmic, and membrane proteins, receptors	mRNA, miRNA, other non-coding RNAs, ssDNA, dsDNA, mitochondrial DNA, cytoplasmic, and membrane proteins
Markers	Anexin V, Flotillin-2, selectin, Integrin, CD40 metalloproteinase	CD63, CD9, Alix, TSG 101, HSP70
Isolation method	Ultracentrifugation (10,000–60,000 g)	• Immunoprecipitation (ExoQuick™) • Ultracentrifugation (100,000–200,000 g) • Ultracentrifugation with density gradient

Until recently, MVs were thought to be an *in vitro* artifact or considered as a system to eliminate waste products from cells (2, 3). Nowadays, MVs are attracting a lot of interest due to their crucial role in intercellular communication (4).

## Physiological functions of EVs

Many studies have stressed the importance of EVs in the normal activity of the central nervous system (CNS). As a matter of fact, glia and neurons release EVs and, as the current literature points out, they play a pivotal role in many physiological effects in the CNS by maintaining homeostasis, including metabolic support, myelin formation and immune defense (5). EVs have been associated with neurogenesis, modulation of synaptic activity and nerve regeneration. As a matter of fact, during the development of the brain in mice, an intensified production of neuronal stem cells was observed (6, 7). The presence of prominin-1 (CD133), a marker of self-renewal and differentiation, found in the vesicles encouraged some hypotheses on the physiological role of EVs that, by interacting with membrane phospholipids and cholesterol, could activate signal transduction pathways leading to cell differentiation (7). Other researchers have proposed that EVs are able to influence the phenotype of other cells as they carry genetic material encoding transcription factors (8). Moreover, EVs transferring b-galactosidase are also considered as important actors of the axonal guidance process (9). Furthermore, several studies have reported that EVs are able to modulate synaptic activity. Cortical neuron derived exosomes after depolarization have been shown to contain cell adhesion proteins and subunits of neurotransmitter receptors (10). Antonucci et al. (11) confirmed the involvement of microglia-derived EVs in synaptic activity by correlating their presence in the presynaptic space to the increase in the release of excitatory neurotransmitters. The EVs produced by Schwann cells have also been associated with the regeneration of damaged nerves, as they carry ribosome or mRNA useful for the synthesis of proteins necessary for their repair (12).

## Pathological involvement of EVs

Although EVs are also produced in physiological conditions, their number is well known to increase during pathological processes, contributing to the development of cancer, cardiovascular and autoimmune diseases. As far as neurological diseases are concerned, many studies have reported changes related to the number and the function of EVs in some common neurological diseases, as well as stroke and epilepsy (13). Agosta et al. showed elevated rates of MVs of myeloid origin in the cerebrospinal fluids (CSF) of patients affected by Alzheimer's disease (AD) and, therefore, suggested that MVs could be useful markers of microglia activation during neuroinflammation in AD (14). Xue et al. (15) have also reported elevated concentrations of MVs in the plasma of AD patients in correlation with their cognitive decline. Moreover, the involvement of EVs in AD is suggested by the association of Amyloid-beta (Ab) with exosomes and it is highlighted by the accumulation of other exosomal proteins, such as flotillin, in the plaques of AD brains (16). However, to date the role of EVs in AD continues to be debated because, depending on the cell of origin, EVs seem to have a toxic or beneficial effect. Yuyama et al. (17), for example, have suggested a neuroprotective role of EVs in AD. As a matter of fact, EVs could act as scavengers of neurotoxic Ab both either by preventing its aggregations and by inducing its proteolysis (18). Furthermore, the beneficial role of EVs in AD could be explained by the presence of the neuroprotective proteins, such as Cystatin C, inside the exosomes released by mouse primar neurons (19).

As far as Stroke is concerned, many studies have reported an involvement of EVs. Most recently, Nanosight tracking analysis (NTA) utilized to determine EV number in the sera of stroke patients has revealed an increase vs. age-matched controls. Moreover, monocyte-differentiated macrophage cultures challenged with EV fractions were found to be activated to produce proinflammatory interleukins such as TNFa and IL1b (20).

## EVs in multiple sclerosis

In the pathogenesis of MS, EVs show both protective and damaging functions. As a matter of fact, EVs could be considered to have a beneficial effect during neurological processes by restoring trophic factors, removing damaged cells (21), controlling synaptogenesis (22), and monitoring the functional state of synapses (23) as described above. Nevertheless, accumulating data suggest that EVs could be implicated in the pathogenesis of MS, especially during relapses involved in cellular activation, facilitating the crossing of the blood–brain barrier and, in this way, amplifying inflammation in the CNS (24).

In 1989 Scolding et al. (25) had already published finding the presence of vesicles in the CSF of subjects suffering from Multiple Sclerosis (MS), suggesting their contribution to myelin damage *in vivo*. More recently, Verderio et al. confirmed these data finding an overproduction of EVs in CSF in subjects with a diagnosis of CIS or MS vs. healthy controls (26). This considerable amount of EVs in CSF was reflected in the remarkable quantity of EVs released by the other cellular components of the blood such as monocytes, leukocytes and platelets, evaluated in MS patients compared to HD (27). Several independent studies report increased levels of EVs in MS patients compared to healthy donors and many authors have measured plasmatic MVs in order to assess a possible correlation between their blood concentration with clinical outcomes and other laboratory tests, as reviewed by Furlan's group (28). In many works these MVs were especially of endothelial origin and associated to the relapsing phase of the disease (29–31). Marco-Ramiro et al. demonstrated a relevant increase in the plasma microparticles coming from the platelets and the endothelium in all clinical forms of MS and in CIS patients (32). Sáenz-Cuesta et al. (27) also extended microvesicle production to platelets, lymphocytes and monocytes. Very recently, a nanoplasmonic approach has confirmed that a significant increase in plasmatic EVs distinguishes CIS and MS patients from controls (33). Moreover, in many cases MVs were used in MS as biomarkers of therapeutic efficacy. The reduction of endothelial MVs has been associated to IFN-β 1b administration (31, 34). Furthermore, Lowery-Nordberg et al.'s prospected study (35) establishes a considerable relationship between the diminished number of MVs and the improvement of the lesions evaluated by MRI. On the contrary, a recent study by Sáenz-Cuesta et al. (27) reported higher levels of plasmatic MVs despite IFN-β and Natalizumab treatment. Dawson et al. proved that Fingolimod suppressed the enzymatic activity of SMase (36), which represents an important step for the MV release. Verderio et al. (26), when observing that EAE mice treated with Fingolimod displayed a reduction in CSF myeloid MVs to baseline levels, hypothesized that the drug might prevent myeloid cell-derived MV shedding from reactive microglia inhibiting the spreading of inflammatory signals throughout the brain.

Although the exact mechanism underlying MV shedding is not yet clearly elucidated, the role of P2X7 receptor (P2X7R) is well recognized. Growing scientific evidence shows that this purinergic receptor, expressed especially on cells of hematopoietic origin (37, 38), is involved in the release of MVs by immune cells especially of the myeloid lineage. As a matter of fact, upon activation of P2X7R by its endogenous ligand ATP or by the synthetic analog Benzoylbenzoyl-ATP (Bz-ATP), microvesicles shedding from the surface of microglia were observed associated with the release of the inflammatory cytokine IL-1B (39).

Very recently, we have reported an elevated amount of microvesicles produced by monocytes of patients affected by MS compared to healthy donors (40). We also observed that treatments currently used in MS affected monocyte-derived microvesicle production. In particular we reported the effects of IFN-β1a and Teriflunomide as first-line treatments able to reduce MV shedding and IL1b gene expression directly related to the duration of the therapy. Considering Fingolimod as a second-line drug, after 12 months of administration, we found the same effect in decreasing MV release.

A short time ago, Furlan et al. demonstrated in the EAE animal model of MS the capability of some interleukins to enhance the production of MVs from cells of myeloid origin without the activation of P2X7R by ATP, suggesting the existence of an alternative pathway that might trigger MV shedding during chronic phlogistic processes (41).

**Figure 1:**
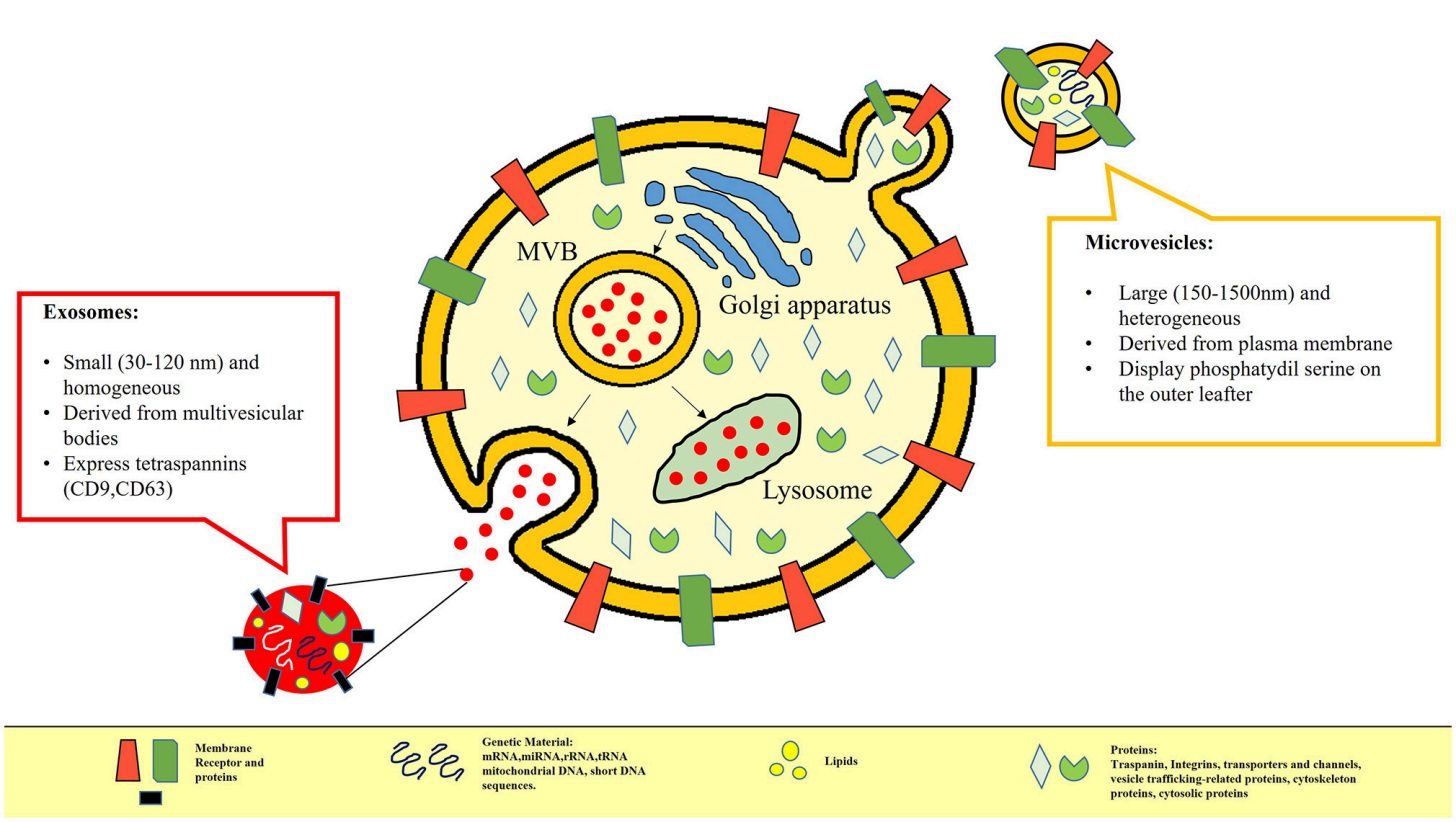
In this picture the differences between MVs and EXOs in terms of different size, biogenesis and content are represented. MVs are small vesicles (100–1,000 nm) which bud directly from the cell plasma membrane and are released into the extracellular environment; EXOs (40–100 nm) are defined as small membrane vesicles formed by inward budding of endosomal membranes called multivesicular bodies that, when fusing with the plasma membrane, release the content in the extracellular compartment. Apart from having different surface markers, Extracellular Vesicles are also considered to be storage pools of diverse bioactive molecules. Their content may include proteins (e.g., signaling molecules, receptors, integrins, and cytokines), bioactive lipids, nucleic acids (e.g., miRNA, mRNA, DNA) and organelles.

In recent years several studies have been focused on the evaluation of MV contents. Despite the increasingly recognized relevance of EVs, the content of EVs still remains little known. The different elements inside the EXOs or MVs could influence their biological and immunological effects. Apart from having different surface markers, EVs are thought to contain different bioactive molecules (42–44), including lipids, proteins such as cytokines, receptors, signaling molecules, and integrins as well as nucleic acids and organelles (7, 45, 46), as shown in Figure 1. In many studies various forms of RNA appear to be enclosed in EVs (47, 48) or conjugates with lipoprotein (49, 50) as a form to prevent degradation. For example, miRNA circulating in blood or present in saliva is reported to be incorporated in EXOs (51). EVs, in particular MVs, because of their characteristic of transferring genetic material (52–54), are suspected of being key actors in intercellular communication and in pathogenesis of the disease. In fact, mRNA encapsulated in MVs has been detected in healthy as well as in diseased subjects (55, 56). Patz et al. showed that CSF-derived MVs could regulate neuronal processes by shuttling mRNA, miRNA, and proteins to target cells (57). To date, the role and the contents ofboth EXOs and MVs combined have still been little explored. There are only a few published studies on evRNAs based on array analysis techniques. For example, Valadi et al., performing a microarray assessment exclusively on EXOs produced by murine or human mast cells, observed the presence of mRNA and small RNA related to 1,300 genes, many of which were not present in the cells which the exosome came from Valadi et al. (58). In contrast, until now the advanced high-throughput next-generation sequencing technique (HT-NGS) has only been applied to the limited field of cultured cells, human urine and circulating RNA (59). Nowadays, a lot of experimental evidence is emerging. Very recently, using this technique, Liguori et al. were able to identify circulating miRNA up regulated and down regulated in pediatric MS contributing to a better understanding of the genetic basis of the disease (60). Also recently, data acquired through NGS analysis have led to the global RNA profile of serum being defined and exosomal miRNA being discovered as a potentially useful biomarker to distinguish MS relapse (61). Actually, thanks to their small volume, MVs may spread from the release site and be found in different biological liquids such as serum, urine, synovial and CSF. Peripheral blood is a great source of EVs (62) and certainly more accessible than CSF. On this basis, performing some preliminary experiments aimed at developing the technique, we analyzed RNA isolated from EVs circulating in serum of a small group of CIS patients (63), obtaining interesting evidence that contributes to improving the knowledge about EV function. In a small cohort of subjects, we were able to identify distinct classes of ncRNAs in both the EV subsets, in line with previous data (58, 61, 62, 64). Furthermore, we found some peculiar differences between CIS and healthy donors that may be considered for use in discriminating between the two conditions. It is worth noting that in depth analysis revealed the most representative ncRNAs classes. In addition, the small expression of rRNA found in MVs could be considered distinctive compared to EXOs, characterized by a higher amount of rRNA, as previously published (59). Interestingly, we detected snRNA in both MVs and EXOs, long ncRNA (lncRNA) fragments, and long intergenic ncRNA (lincRNA) fragments in line with the cytoplasmic origin of EVs, considering that RNA degradation occurs in the cytoplasm (65).

Ebrahimkhani et al. (66), using NGS, have also identified differentially expressed exosomal miRNAs extracted from sera samples in both RRSM and progressive MS patients compared to healthy donors. Indeed, they report dysregulated miRNA that can reasonably distinguish RRSM or SPMS and PPMS patients from healthy controls. It is worth noting that they identified nine miRNA that may help to discriminate between the stages of the disease, providing a useful tool in assisting the determination of the transition from RRMS to SPMS. To date, there are no clinical parameters that allow the passage from one stage to another of the disease to be predicted. Therefore, these findings, if confirmed by longitudinal studies, may well have clinical and economic implications.

## Open issues

Despite the introduction of cutting-edge approaches for their characterization, current knowledge on EVs is still inadequate to provide exhaustive information about their morphology, size, density, phenotype, concentration, and content. The sub-micrometer size of EVs and the intrinsic limitations in methods of characterization of EVs make the development of standardized protocols of sample preparation and of EV measurement conditions very difficult. For example, there is little agreement concerning the concentration of EVs in plasma: values ranging from 200 to 10^9^ EVs uL^−1^ are reported in the literature (67), hence several orders of magnitude of difference according the method utilized for detection. To date, the various research groups have used different procedures both for the isolation and the analysis of EVs as summarized in Table 2 and in Table 3. Recently, Coumans et al. (68) tried to draw up a methodological guideline to study EVs by providing an expert-based update of recent advances in the protocols and by summarizing currently accepted considerations and recommendations in every stage of analysis from sample collection to extraction, detection, and characterization of EVs. This remains a crucial point that needs to be addressed in future in order to establish internationally recognized criteria for the objective evaluation of the roles of EVs in health and disease, as well as their biomedical potential.

**Table 2:** Commonly used methods for isolating EVs.

**Methods for isolating EVs**			
**Method**	**Description**	**Advantages**	**Disadvantages**
Differential centrifugation	Low-g force (500–2,000 g) centrifugation step for removing cell and debris followed by low–g force spin (10–20,000 g) to isolate MVs and subsequently followed by centrifugation at 100,000 g for EXOs	Easy to use; low-cost	Low-purity (contaminant: protein oligomers/protein-RNA complexes and viruses other particles with similar size and density), time consuming, requires expensive lab. equipment such as an ultracentrifuge
Density gradient centrifugation	Fractionates EVs on the basis of buoyant density using a discontinuous gradient of a sucrose solution or less-viscous iodoxinol	High purity	Lower yield due to sample loss during centrifugation
Immunomagnetic bead	Based on magnetic beads coated with antibodies against specific exosomal markers such as the tetraspanins C9 or CD81	Easy to use; specificity; high purity	Commercial kits available only for exosomes; prior knowledge of EVs required
Affinity purification chromotography	Relied on affinity tag such as monoclonal antibody that target specific antigens expressed on the surface of EVs	Specificity; high purity	Low yield; prior knowledge of EVs required
Precipitation	Based on several synthetic water-soluble polymers, commonly used as protein/virus/particle precipitants, are used to rapidly isolate EVs	High yield	Low purity due to coisolation of proteic contaminants. Requires pre-and post-cleanup
Size-based techniques: Ultrafiltration and SEC	Exosome isolation is exclusively based on the size difference between exosomes and other particulate constituents	Ultrafiltration: Fast, does not require special equipment, direct RNA extraction possible. SEC: high-purity exosomes, gravity flow preserves the integrity and biological activity; superior reproducibility	Ultrafiltration: low equipment cost, moderate purity of isolated exosomes, shear stress induced deterioration, possibility of clogging and vesicle trapping, exosomes loss due to attaching to the membranes. SEC: Moderate equipment cost, requires dedicated equipment, not trivial to scale up, long run time

**Table 3:** Main techniques for EV analysis.

**Methods for quantifying EVs**			
**Method**	**Description**	**Advantages**	**Disadvantages**
Nanoparticle tracking (NTA)	Tracks the Brownian motion of the particles in scattering or in fluorescence mode and and by measuring the scattering intensity of single particles infers their size	Accurate for both monodisperse and polydisperse; calibration particle standards	Size >70 nm; requires specific instruments (Nanosight and Zetaview)
Dynamic light	Analysis the fluctuations of scattering intensity of particles in Brownian motion	Accurate for monodisperse samples; lower size (< 30 nm)	Large particles can compromise the results, inaccurate for polydisperse samples; specific instrument required (NanoZS and Nanoflex); requires a high concentration of monodisperse particles to be detected, which is not convenient for low yields of collected EVs
Resistive pulse sensing	Measures the change in conductance across a sensing pore upon passage of a particle	Surface charge	For unknown size distribution, insufficient for detection of all particles, size >70 nm. Requires specific instruments : qNano
Flow cytometry	Measures scattering or fluorescence intensity of particles illuminated by a laser	Low particle concentration (106 particles ml^−1^)	Size >200 nm. For EXOs not absolute size measurement. A flow cytometer required
Electron microscopy: cryo-EM (cryoelectron microscopy)and TEM (transmission electron microscopy)	Utilizes electrons instead of photons to create an image with a resolution down to the nanometer	TEM/cryo-EM: direct visualization and observation of EVs, EV structure/morphology; cryo-EM: preserves membranes in native state	TEM: fixation induces shrinking of EV structure, equipment: electron microscope, cost
Immunoblotting (IB)	IB is based on the detection and relative quantification of EVs by using specific antibody against characteristic markers such as CD9, CD63, CD81, TSG101, Alix, actin, tubulin, flotillin-1, HSC70/HSP73, HSP70/HSP72, and MHC molecules	In combination with other techniques IB is largely used to characterize and assess the degree of purity of EV preparations: the absence of cell-derived organelle markers such as calreticulin is often used to assess the purity of an EV preparation	IB cannot be used to quantify EVs and the enrichment of these proteins in the EV fraction does not guarantee the absence of contaminants

## Conclusions

In conclusion, this review summarizes the state of the art on extracellular vesicles in MS. Long gone are the times when EVs were considered as simply scrap products. Nowadays they are known to be important players in physiological processes and in the pathogenesis of many diseases. Although a lot of effort has been made and yet more is still ongoing contributing to the significant progress on the physical and biological characterization of EVs, many aspects still remain not fully elucidated and certainly deserve to be addressed in the near future. Overall, recent scientific evidence seems to be highly promising and strongly encourages more in-depth studies to further explore the potential of EVs as biomarkers in monitoring disease course and response to treatment.

## Author contributions

MB and CA defined the research questions and aims of the study. AA, TM, and MB carried out the literature search and selected relevant papers. MB wrote the first draft of the manuscript. CA revised and corrected the manuscript. All authors read and approved the final manuscript.

### Conflict of interest statement

The authors declare that the research was conducted in the absence of any commercial or financial relationships that could be construed as a potential conflict of interest.
